# Survival Prediction of Patients with Bladder Cancer after Cystectomy Based on Clinical, Radiomics, and Deep-Learning Descriptors

**DOI:** 10.3390/cancers15174372

**Published:** 2023-09-01

**Authors:** Di Sun, Lubomir Hadjiiski, John Gormley, Heang-Ping Chan, Elaine M. Caoili, Richard H. Cohan, Ajjai Alva, Vikas Gulani, Chuan Zhou

**Affiliations:** 1 Department of Radiology, University of Michigan, Ann Arbor, MI 48109, USA; lhadjisk@med.umich.edu (L.H.); jrgorm@umich.edu (J.G.); chanhp@med.umich.edu (H.-P.C.); caoili@med.umich.edu (E.M.C.); rcohan@med.umich.edu (R.H.C.); vikasgulani@med.umich.edu (V.G.); chuan@med.umich.edu (C.Z.); 2 Department of Internal Medicine-Hematology/Oncology, University of Michigan, Ann Arbor, MI 48109, USA; ajjai@med.umich.edu

**Keywords:** bladder cancer, survival prediction, radical cystectomy, nomogram, radiomics, deep learning

## Abstract

**Simple Summary:**

Survival prediction of bladder cancer patients following cystectomy is essential for treatment planning. We propose a hybrid method that integrates clinical, radiomics, and deep-learning descriptors to improve survival prediction models. This approach demonstrates potential for more accurately predicting survival and prognosis in radical cystectomy treatment and in determining whether imaging adds additional predictive value over patients’ clinical information.

**Abstract:**

Accurate survival prediction for bladder cancer patients who have undergone radical cystectomy can improve their treatment management. However, the existing predictive models do not take advantage of both clinical and radiological imaging data. This study aimed to fill this gap by developing an approach that leverages the strengths of clinical (C), radiomics (R), and deep-learning (D) descriptors to improve survival prediction. The dataset comprised 163 patients, including clinical, histopathological information, and CT urography scans. The data were divided by patient into training, validation, and test sets. We analyzed the clinical data by a nomogram and the image data by radiomics and deep-learning models. The descriptors were input into a BPNN model for survival prediction. The AUCs on the test set were (C): 0.82 ± 0.06, (R): 0.73 ± 0.07, (D): 0.71 ± 0.07, (CR): 0.86 ± 0.05, (CD): 0.86 ± 0.05, and (CRD): 0.87 ± 0.05. The predictions based on D and CRD descriptors showed a significant difference (p = 0.007). For Kaplan–Meier survival analysis, the deceased and alive groups were stratified successfully by C (p < 0.001) and CRD (p < 0.001), with CRD predicting the alive group more accurately. The results highlight the potential of combining C, R, and D descriptors to accurately predict the survival of bladder cancer patients after cystectomy.

## 1. Introduction

Bladder cancer is the tenth most common cancer worldwide [1], and the fourth most common cancer in men [2]. For muscle-invasive and recurrent non-invasive bladder cancer, radical cystectomy is a crucial treatment [3,4]. The procedure involves the complete removal of the bladder. In men, it also typically includes the removal of the prostate and seminal vesicles; in women, it often includes the removal of the uterus, ovaries, fallopian tubes, and part of the vagina [5].

The five-year survival rate serves as a valuable indicator of treatment effectiveness, varying across different types of cancer. Testicular cancer (97%), melanoma of the skin (92.3%), and prostate cancer (88%) have the highest estimated five-year survival [2]. Conversely, lung and bronchial cancer exhibit the lowest survival rate at only 22% [2]. In the United States, the five-year relative survival rate for bladder cancer stands at 77% [2]. Specifically, for patients with bladder cancer who underwent radical cystectomy, the reported five-year survival rates range from 54.5% to 68% [6].

Survival prediction in bladder cancer can be performed based on data sources such as clinicopathological information [7], histological slides [8,9], gene expression, or molecular markers [10]. The analysis methods include machine-learning models like support vector machines, logistic regression, and random forest [11,12], as well as nomogram [13] and risk stratification [14]. Nomogram models, often developed based on large-size cohorts, can offer high-accuracy prediction capabilities.

CT urography (CTU) plays a significant role in bladder cancer diagnosis by providing 3D abdominal images that allow for the quantitative volumetric analysis of the bladder and lesion characteristics. The recent advances in deep-learning models offer new opportunities for extracting more comprehensive image features and the potential for improving the predictive models.

In this study, we combine clinical, histopathological features with CTU image features to harness the advantages of nomogram, radiomics, and deep-learning models. By introducing a hybrid approach combining clinical and imaging analytics, we aim to enhance the prediction of five-year survival for patients with bladder cancer after radical cystectomy, allowing better treatment planning and improving prognostic outcomes. The approach also has implications for the assessment of the added value of imaging in patient management and for how the added predictive value could be used to select the utilization of imaging.

## 2. Materials and Methods

**Figure 1:**
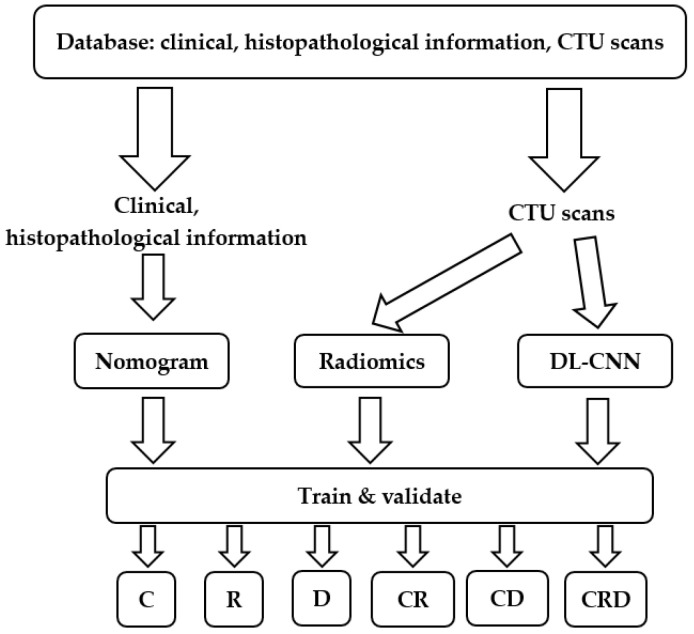
Predictive model development process of this study. We collected clinical, histopathological information and CTU scans. We built and validated survival prediction models based on individual or combined descriptors. C = clinical descriptors; R = radiomics descriptors; D = deep-learning descriptor; CR = clinical + radiomics descriptors; CD = clinical + deep-learning descriptors; CRD = clinical + radiomics + deep-learning descriptors. DL-CNN = Deep learning–convolutional neural network.

The model development process is depicted in Figure 1. First, we collected a comprehensive dataset comprising clinical, histopathological information and CTU scans. One pair of pre- and post-treatment CTU scans was obtained for each patient. We then processed the collected data and developed models incorporating nomogram, radiomics, and deep-learning descriptors. The models were built with a training set and optimized with a validation set. After the models were developed, they were deployed to an independent test set to evaluate their survival prediction ability on unseen cases.

### 2.1. Patient Cohorts

With Institutional Review Board approval, histopathological information and CTU scans were collected for patients who had been diagnosed with bladder cancer. Patients were selected if they met all of the following criteria: (1) patients who underwent neoadjuvant chemotherapy and had at least one CTU scan before chemotherapy and one after chemotherapy; (2) patients who underwent radical cystectomy; (3) patients for whom follow-up information after surgery was available to determine their survival status.

**Figure 2:**
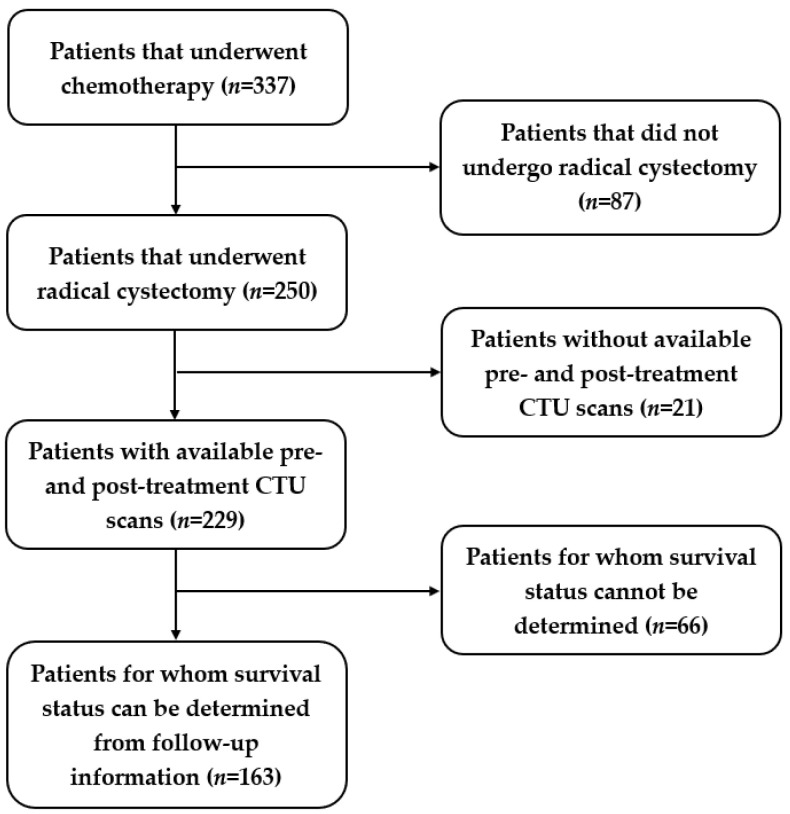
Study-population diagram. Out of 337 patients, 163 patients were identified for this study.

We identified a total of 163 patients who satisfied the above criteria out of 337 patients. The study-population flow diagram is shown in Figure 2. Among the 163 patients, 79 patients were alive at the 5-year mark after receiving radical cystectomy, while 84 patients died before reaching the 5th year after cystectomy treatment. We split the data into three sets: 56% (92/163) training (55 alive; 37 deceased); 4% (7/163) validation (4 alive; 3 deceased); and 40% (64/163) test (20 alive; 44 deceased). We used a serial approach to assign the cases to the training, validation, and test datasets. We aimed at keeping a relatively large portion of the patients in the training and test sets. The bladder cancer cases were collected chronologically. The exam dates of the cases in the training and validation sets ranged from 2006 to 2012, and the cases in the test set ranged from 2015 to 2020. The serial approach simulates to some extent the “real world” clinical situation, where the model is built based on previous cases; after all weights and parameters are fixed, the model is applied to the new incoming cases.

The collected clinical and histopathological information included five indices: post-surgery pathologic stage, lymphovascular invasion (LVI), pathologic node stage, whether patients underwent neoadjuvant chemotherapy, and whether patients underwent adjuvant radiotherapy. Chemotherapy plays a significant role in bladder cancer treatment influencing the survival of patients. The pre- and post-treatment CTU scan pairs were analyzed for changes in image features related to treatment response that are useful for survival prediction.

### 2.2. Nomogram

The histopathological information was analyzed using the nomogram developed by Shariat using a cohort of 731 patients [15]. The nomogram achieved a prediction accuracy of 0.791 in the study by Shariat [15], which was superior to that of the American Joint Committee on Cancer Staging [16] at 0.663 with *p* = 0.001.

The five clinical and histopathological indices described above were used as the input to the nomogram. For every patient, each of the five inputs was mapped to a “points” axis to determine how many points were contributed by each input. The sum of the points from the five inputs resulted in the estimation of the “total points” for the patient, which was then mapped to the *n*-year survival probability (*n* = 5 in the current study).

### 2.3. CTU Scans Processing

**Figure 3:**
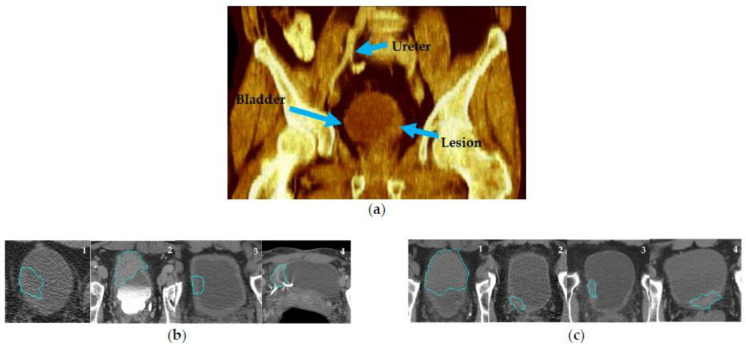
CTU scan examples. (**a**) A 3D volume rendering CTU scan showing the ureter, bladder, and bladder lesion. (**b**) CTU scans that are potentially challenging for lesion segmentation. (**b**)-1: without contrast; (**b**)-2: delayed-contrast phase; (**b**)-3: bladder wall thickening; (**b**)-4: image artifact. (**c**) Examples of CTU scans in early contrast phase and without artifacts. The blue outlines are the AI-CALS segmentation outlines.

CTU scans for bladder cancer include a series of abdominal images, allowing for a comprehensive 3D inspection of the bladder. Figure 3a shows an example of a rendered 3D CTU scan. To extract radiomics and deep features from the CTU scans, the bladder cancer lesions were annotated by an experienced radiologist (R.H.C.) who has over 30 years of experience reading abdominal CT. The annotation process entailed several steps: (1) marking a volume of interest (VOI) for the lesion, (2) measuring the longest and perpendicular diameters of the tumor, (3) identifying the lesion location within the bladder, ureter, or other abdominal locations, and (4) evaluating the lesion type, edge characteristics, and likelihood of abnormality. While a patient could have multiple lesions, for this study, we limited the number of lesions to one per patient and selected the dominant lesion for feature extraction.

**Figure 4:**
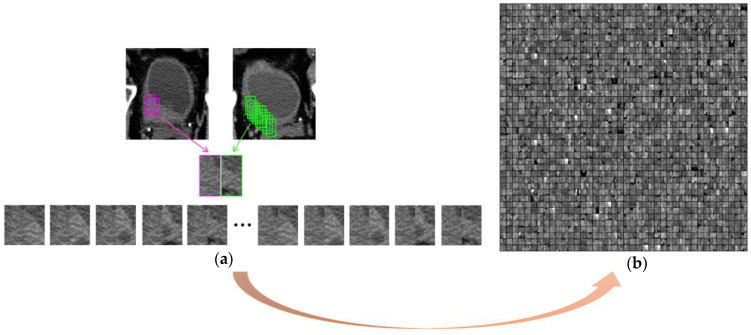
Formation of hybrid ROIs. (**a**) Use a sliding-window technique to extract ROIs from the VOI. A hybrid ROI is formed by combining one ROI from the pre-treatment scan on the left side (pink frame) and one ROI from the post-treatment scan on the right side (green frame). Different combinations result in a number of hybrid ROIs from one pre- and post-treatment scan pair. (**b**) A subset of hybrid ROIs of the training set shown in a matrix. Each hybrid ROI (32 × 32 pixels) was a separate sample during deep-learning model training.

Lesion segmentation was obtained by our in-house developed semi-automatic algorithm AI-CALS [17] utilizing the marked VOIs. The segmentation provided 3D contours outlining the lesion, from which we performed feature extraction. In a previous study, we showed that the AI-CALS algorithm achieved an average volume error of 4.9 ± 38.3% when compared to the manual outlines drawn by an experienced radiologist [17]. Segmenting lesions in CTU scans can be challenging due to various factors, such as patient positioning, image artifacts, and excreted contrast in the bladder. Ideally, since bladder cancer is expected to enhance in the early contrast phase, these images can help to distinguish neoplasm from the rest of the bladder tissue. However, some patients underwent CTU with different imaging protocols from referral sites including only non-contrast images (Figure 3b-1) for various reasons such as renal failure or patient refusal of contrast. Other protocols consisted of only delayed contrast phase imaging (Figure 3b-2) which could decrease the conspicuity of the lesion from surrounding structures. Figure 3b-3 shows a case with bladder-wall thickening after treatment, in which it is difficult to ascertain whether the lesion still existed. Additionally, artifacts in CTU scans can also complicate segmentation (Figure 3b-4). Despite these challenges, the performance of AI-CALS was satisfactory in most cases. Figure 3c illustrates CTU examples with early contrast phase and minimal artifacts, for which the segmentation was in good agreement with the manual reference.

### 2.4. Radiomics Model

Radiomics features derived from medical images have the potential to reveal intricate tumoral patterns and characteristics that may not be perceivable by the naked eye. The radiomics features (RF) developed in our group [18,19,20] demonstrated a good performance across various tasks, including lung nodule classification [21], mammographic mass characterization [22], and bladder cancer treatment response assessment [23].

A set of 91 RF features was extracted, including morphological, texture, and intensity-based features. Thus far, these features are primarily limited to 2D analysis. In this study, we extracted RF features from the central slice of the lesion, including the RF features from pre-treatment scans, referred to as $f_{pre}$, and the RF features from post-treatment scans, referred to as $f_{post}$. To capture the changes in features between pre- and post-treatment scans, the difference features $f_{diff}$ were calculated by:

(1) $$f_{diff}=\frac{f_{pre}-f_{post}}{f_{pre}},$$

We therefore obtained a total of 273 radiomics features. Feature selection can eliminate the redundant variables, reduce the time and space requirements for data processing, and reduce the risk of the “curse of dimensionality” when the training data size is limited [24,25,26]. We employed a mutual information (MI) method for feature selection.

MI is capable of measuring the arbitrary dependencies between random variables and has been widely used for feature selection in machine learning [27]. One of the advantages of MI is its ability to assess the information content of features in nonlinear relations. Equation (3) shows that MI is formulated as the difference in the entropies between the variable itself (Equation (2)) and the variable conditioned on the target value. The MI score ranges from 0 to ∞, where zero signifies complete independence between the feature and the target, while higher scores indicate greater relevance such that the corresponding feature may be useful for the learning task.

For a feature vector f, the entropy is:

(2) $$H\left(F\right)=-\int P\left(f\right)logP\left(f\right)df$$

Given the condition of C, the MI between variables c and f is:

(3) $$I\left(F;C\right)=H\left(F\right)-H(F|C)$$

In order to select the useful set of features, a threshold was imposed on the calculated MI. Pearson correlation was applied to estimate the feature correlation.

### 2.5. Deep-Learning Model

To extract deep features from the paired pre- and post-treatment scans, we employed a hybrid-ROI strategy [28]. This approach, illustrated in Figure 4a, involved creating a hybrid-ROI that is composed of one ROI of size 32 × 16 pixels from the pre-treatment scan on the left side and one ROI of the same size from the post-treatment scan on the right side. The resulting hybrid ROI has a size of 32 × 32 pixels. We utilized a sliding-window technique to extract the ROIs from the VOI. To ensure a balanced dataset and prevent dominance or bias caused by CTU scan pairs with large lesions, we imposed a threshold to limit the number of hybrid ROIs from one scan pair. All hybrid ROIs from the same case were assigned the same case label, survived over 5 years = 1, otherwise = 0. A subset of hybrid ROIs from the training set is shown in Figure 4b.

**Figure 5:**
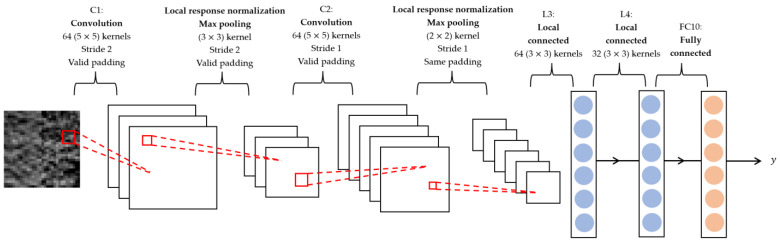
Architecture of CNN. The input image has a size of 32 × 32 pixels. The CNN was composed of two convolution layers (C1 and C2), each of them was followed by a local response normalization layer and a max-pooling layer, two locally connected layers (L3 and L4), and one fully connected layer (FC10). The kernel, stride, and padding setting of each layer are illustrated. The output is a likelihood score.

Deep features were extracted from the hybrid ROIs by a Convolutional Neural Network (CNN) [29]. The structure of the CNN is depicted in Figure 5, comprising two convolutional layers, C1 and C2, each of which is accompanied by a local response normalization layer and a max-pooling layer; two locally connected layers, L3 and L4; and a fully connected layer, FC10. The C1, C2, and the max-pooling layers could be considered as a deep-feature extractor. The last three fully connected layers synthesized the deep features into a likelihood score for each input image (hybrid ROI). The likelihood scores of all the hybrid ROIs from one patient were combined into a single likelihood score that reflected the survival likelihood of that patient.

### 2.6. Classification

**Figure 6:**
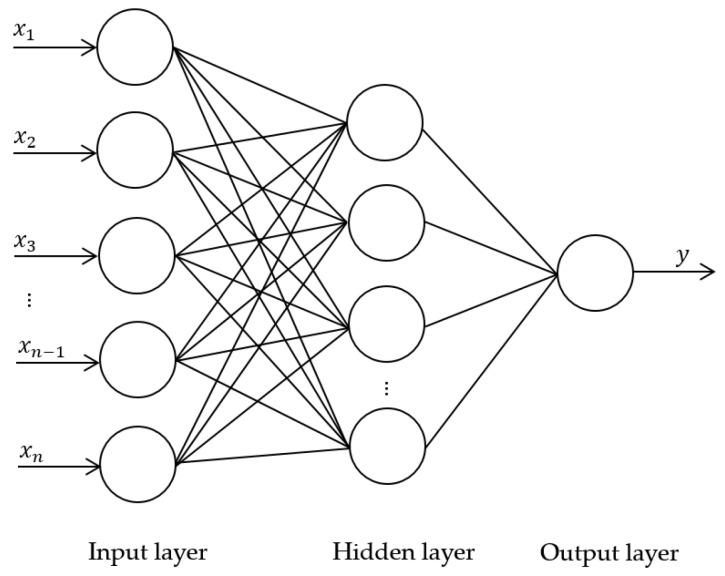
Structure of BPNN. The input $x_{i}$ (i=1, 2, 3, ..., n) are the descriptors. The hidden layer contains 13 nodes. The output y is the likelihood score assessing the survival of each patient.

The features from the three types of descriptors of each case, i.e., the selected radiomics features by the MI, the survival likelihood score from the deep-learning model, and the “points” derived from each of the five indices of the nomogram, were fed into a Back-Propagation Neural Network (BPNN) [30] to classify the likelihood of survival of each patient. The performance of the descriptors when they were used individually was compared to the utilization of the descriptors in combination. The structure of the BPNN is shown in Figure 6, where the parameters of the BPNN were selected as guided by the validation set.

### 2.7. Statistical Analysis

The classification performance of the descriptors was evaluated using the area under the receiver operating characteristics (ROC) curve (AUC). We compared three combinations of descriptors for survival prediction (C vs. CRD, R vs. CRD, and D vs. CRD); thus, the critical α value for statistical significance was adjusted to be α = 0.017 (α = 0.05/3) according to the Bonferroni correction for multiple hypothesis testing [31,32].

To further analyze the model performance on predicting survival outcomes, we conducted a Kaplan–Meier analysis based on a log-rank test [33] to generate the survival curves.

## 3. Results

### 3.1. Cohort Statistics

**Figure 7:**
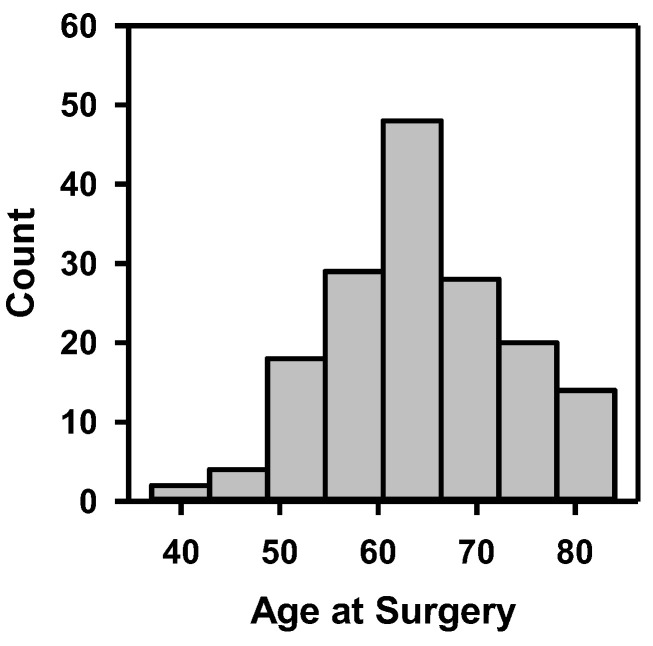
Histogram of patients’ age at surgery.

The demographic information and cancer stage of the 163 patients are shown in Table 1, including gender, race, tobacco use, clinical stage of cancer, and pathological stage of cancer after radical cystectomy. The distribution of patients’ age at surgery is depicted in Figure 7. These statistics show that the dataset covered a wide range of patient demographics.

### 3.2. Radiomics Features

The MI-selected radiomics features were evaluated by Pearson correlation on the training set. First, we compared the feature vector with the target vector. In our analysis, we aimed for a strong correlation between feature and target, as this would allow us to predict the target from the feature. Then we compared the feature vectors among themselves. It is preferable to have the selected features less correlated to make the model more robust. However, features with some correlation as a group may also be useful in the model design.

**Figure 8:**
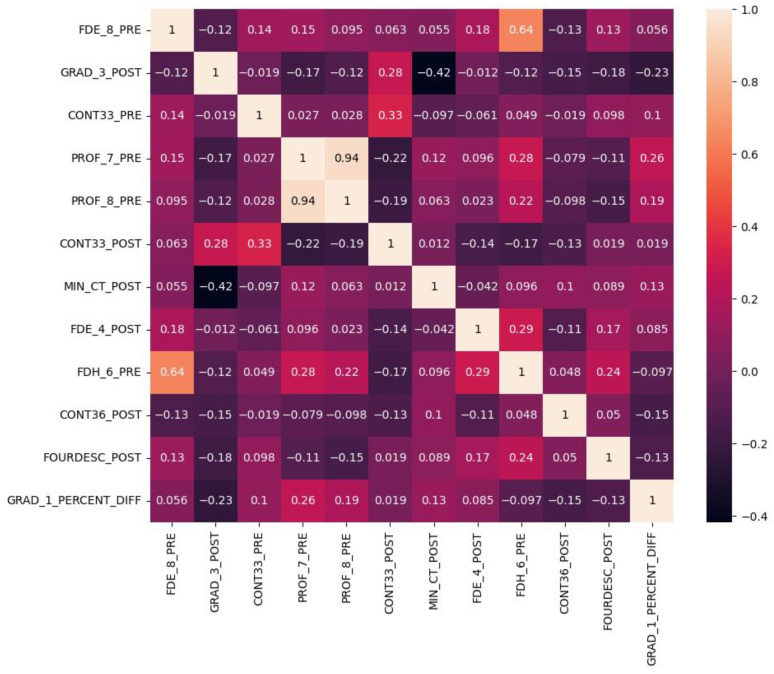
Heatmap of Pearson correlation between any pair of the MI-selected radiomics features. Most of the correlation values were within ±0.3 which indicates a weak correlation.

Out of the 12 selected features, we plotted a heatmap showing the correlation values between each pair of features (Figure 8). The majority of the correlation values fell within the range of ±0.3, indicating weak correlations. Weak correlations implied that the selected features were providing mostly distinct and independent information, which can contribute to the predictive power of the model with a low redundancy. On the other hand, the features with a certain level of correlation may contribute to the predictive power when combined with other features.

### 3.3. Survival Prediction

**Figure 9:**
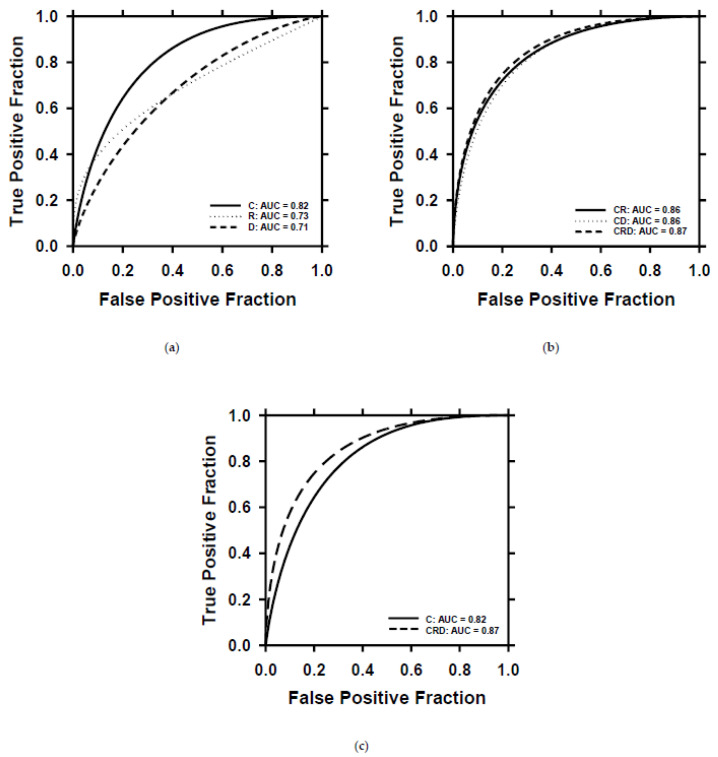
ROC curves of survival prediction on the test set by using different descriptors. (**a**) ROC curves and AUC values for the individual descriptor. (**b**) ROC curves and AUC values for combined descriptors. (**c**) Direct comparison of the ROC curves of C and CRD descriptors. C = clinical descriptors; R = radiomics descriptors; D = deep-learning descriptor; CR = clinical + radiomics descriptors; CD = clinical + deep-learning descriptors; CRD = clinical + radiomics + deep-learning descriptors.

After the training of the models with the training set, we selected the best-performing clinical, radiomics, deep-learning, and combined models using the validation set. For the combined models, BPNN training required about 40 iterations. We deployed the selected models on the test set to evaluate their ability to predict patient survival. The ROC curves of the individual and combined descriptors are plotted in Figure 9. The clinical (C), radiomics (R), and deep-learning (D) descriptors achieved AUCs of 0.82 ± 0.06, 0.73 ± 0.07, and 0.71 ± 0.07, respectively. By combining the R and D descriptors with the clinical C descriptors, the AUCs of the models were improved to 0.86 ± 0.05 for CR, 0.86 ± 0.05 for CD, and 0.87 ± 0.05 for CRD. The ROC curves of C and CRD are plotted in one figure (Figure 9c) to show their differences in classification performance. The classifications based on D and CRD descriptors achieved a significant level of difference (*p* = 0.007) (Table 2). Other pairs of comparisons did not reach statistical significance but showed strong trends towards improvement when used in combination.

**Figure 10:**
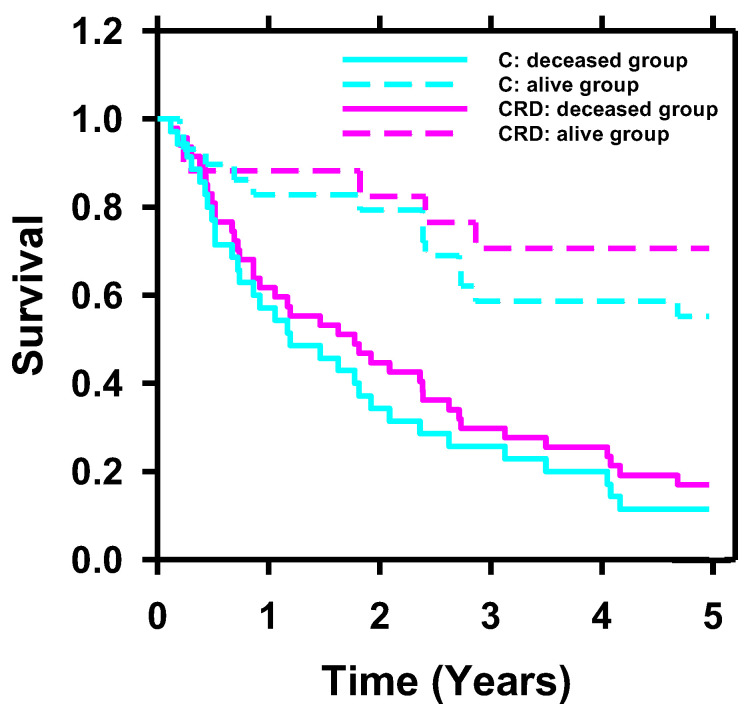
Kaplan–Meier survival probability analyzed with C and CRD descriptors. Stratification of the two groups (deceased and alive) achieved statistical significance using C (*p* < 0.001) and CRD (*p* < 0.001) descriptors. For the alive group, the survival curve of CRD was superior to that of C.

To further investigate the impact of incorporating radiomics and deep-learning descriptors into clinical descriptors on survival prediction, we conducted Kaplan–Meier analysis on C and CRD descriptors. To categorize the patients in the test set into two groups (deceased and alive groups), we determined the cutoff values of the scores generated by BPNN for C and CRD descriptors by selecting the point of least misclassification for the C and CRD models separately based on the training and validation sets. Figure 10 displays the Kaplan–Meier curves of the deceased and alive groups predicted by C (*p* < 0.001) and CRD descriptors (*p* < 0.001). For the alive group, the CRD-generated curve was superior to the C-generated curve.

## 4. Discussion

All three descriptors—C, R, and D—showed strength in classifying the test dataset. However, the clinical descriptors demonstrated a higher performance compared to the deep-learning and radiomics descriptors. One of the main factors contributing to this result is that the nomogram model utilized in the clinical descriptors was developed using a larger training set, while the deep-learning and radiomics models used here were trained on a smaller dataset. Deep-learning models are known to achieve a better generalization and robustness when they are trained with larger and more representative datasets [34].

With the integration of radiomics and deep-learning descriptors alongside the clinical descriptors, the CRD descriptors can stratify the deceased and alive groups more effectively. The lack of statistical significance in the difference between the AUCs of the C and CRD descriptors, and the modest improvement in the stratification ability in the survival curves between the deceased and alive groups by CRD compared to that of the C descriptors, may be attributed to the small size of the test set, which could limit the statistical power to detect the small differences, and also the strong performance of the clinical descriptors alone. While this is a preliminary study and requires significant further investigation, the result has implications for the utilization of this approach in the future, as it could be used to assess whether an imaging test has added value over clinical data alone. If an imaging test is expected to add predictive value for the clinical task at hand, it will strengthen the utility of the imaging test for patient care.

The results also point to the importance of clinical information in the utilization of imaging, as this information can lead to the improved performance of imaging in answering the clinical question. This is well accepted in clinical radiology, but clinical information is not typically utilized in radiomics or machine-learning computational models for imaging tasks. This study demonstrated that the clinical data may contain important complementary information to improve the performance of these models.

Our study has some limitations. The dataset was relatively small and collected from a single site. We need to collect multisite cases and larger datasets to build more reliable and generalizable models. We also included non-contrast CT images which could affect the performance of the R and D models. On the other hand, the presence of non-contrast CT images represents the real clinical situation and the models may have been trained to be more robust with the heterogeneous image features.

Despite the limitations, this study demonstrated that CTU scans potentially can provide useful information to improve the survival prediction of patients with bladder cancer after radical cystectomy. Radiomics and deep-learning models can extract effective image features from CTU scans. Combined with a nomogram, the prediction ability of the hybrid model can outperform the models using the individual types of descriptors.

## 5. Conclusions

While larger datasets are needed, this study demonstrates that combining radiomics and deep-learning descriptors with clinical descriptors holds promise for improving the prediction of the 5-year survival of bladder cancer patients after radical cystectomy. The accurate assessment of 5-year survival offers potential benefits with patient counseling and postoperative surveillance strategies.

## Figures and Tables

**Table 1:** Patient characteristics (n = 163).

Attributes		#
Gender	Male	131
	Female	32
Race	White	142
	Black/African Am.	13
	Am. Indian/Native	1
	Asian	2
	Unknown	5
Tobacco use	Current	40
	Former	88
	Never	34
	Unknown	1
Post-surgery stage	pT0	35
	pTa/pTi/pTis	15
	pT1	16
	pT2	36
	pT3	45
	pT4	16
Lymphovascular invasion (LVI)	Yes	61
	No	102
Pathologic node stage	N0	112
	N1	24
	N2	23
	N3	4
Neoadjuvant chemotherapy	Yes	163
	No	0
Adjuvant radiotherapy	Yes	0
	No	163

**Table 2:** The *p*-values of survival prediction based on individual and combined descriptors. The critical α value for statistical significance was adjusted to be α = 0.017 (α = 0.05/3) according to the Bonferroni correction for multiple hypothesis testing.

Comparison	*p*-Value (Adjusted α = 0.017)
C vs. CRD	0.153
R vs. CRD	0.056
D vs. CRD	0.007 *

* Achieved a significant difference.

## Data Availability

Data available upon request.

## Abbreviations

Deep learning-convolutional neural network (DL-CNN); Clinical descriptors (C); radiomics descriptors (R); deep-learning descriptor (D); clinical + radiomics descriptors (CR); clinical + deep-learning descriptors (CD); clinical + radiomics + deep-learning descriptors (CRD); CT Urography (CTU); lymphovascular invasion (LVI); volumes of interest (VOI); radiomics features (RF); mutual information (MI); Convolutional Neural Network (CNN); Back-Propagation Neural Network (BPNN); receiver operating characteristics (ROC); area under ROC curve (AUC).
